# Managing paediatric obesity: a multidisciplinary intervention including peers in the therapeutic process

**DOI:** 10.1186/1471-2431-14-89

**Published:** 2014-04-03

**Authors:** Helena Fonseca, António Labisa Palmeira, Sandra Cristina Martins, Liliana Falcato, António Quaresma

**Affiliations:** 1 Paediatric Obesity Clinic, Department of Paediatrics, Hospital de Santa Maria, Lisboa, Portugal; 2 Faculdade de Educação Física e Desporto, Universidade Lusófona, Campo Grande 376, Lisboa, 1749-024, Portugal; 3 CIPER – Faculdade de Motricidade Humana, Universidade de Lisboa, Portugal

**Keywords:** Adolescent, Obesity, Physical Activity, Weight Management, Peers

## Abstract

**Background:**

Adolescent obesity epidemic is one of the major health priorities as it tracks into adult life. There is widespread need for new creative strategies and lifestyle programs. This study was designed to investigate the possible impact of including peers on the weight management program and assess the long-run adherence to behaviour change, with a potential positive impact on body mass index, body composition, and physical activity. Peer influence is major at this age and it is expected that adolescents will be better motivated and engaged in the behaviour changes when they are accompanied by their friends.

**Methods/design:**

The study is a non-randomised, non-blinded controlled trial, including two groups: 1) Comparison group (n = 35), which will receive a 12 month standard treatment at the hospital setting plus a weekly interactive and physical activity session; 2) Experimental group (n = 99), which will receive the standardized treatment at the hospital plus a weekly session together with a peer of their choice. The sample size calculations for the primary outcomes showed that we will have power to detect effect sizes of 0.25. Measures include: a) Dual-energy x-ray absorptiometry (for body composition assessment); b) Anthropometric evaluations; c) Assessment of physical activity levels by accelerometers; d) Psychosocial mediators (motivation and peer support) assessed with a package of psychometric questionnaires; and e) Outcomes (quality of life and well-being).

**Discussion:**

Adolescence is a crucial period for the development of a healthy lifestyle, especially among those who reach this age with an obesity condition. Obesity management programs directed to adolescents are often an adopted version of programs developed for children, most of them with a strong focus on the family, or an adopted version of adult programs, not recognizing the specificities of this age group. This study is designed taking into account the unique characteristics of this life-cycle stage, with the main objective of testing an innovative treatment for adolescent obesity.

**Trial registration:**

This trial is registered in the clinicaltrials.gov with the number NCT02024061

## Background

Paediatric overweight and obesity have reached epidemic proportions in most industrialised countries [1]. In Portugal, a Southern European country with a traditional Mediterranean diet, the prevalence of overweight adolescents is among the highest in Europe. In a recent study [2], using a representative sample of Portuguese young people (22048 adolescents) aged between 10 and 18 years, there was an estimated prevalence of overweight and obesity of 23.1% and 9.6%, among girls and of 20.4% and 10.3% among boys, respectively. It was also seen that the prevalence of overweight and obesity decreases with age for boys and for girls from 13 to 18.

It is of the utmost importance to work on prevention to deal with this epidemic [3,4]. However, efforts must also be made to treat those young people who are already overweight [1,5]. There are several facts to support this need, in particular because adolescent obesity: a) has a significant impact on physical and psychosocial health [6]; b) is an independent risk factor for adult obesity [7]; c) is an independent risk factor for mortality in adults, perhaps more powerful than adult obesity itself [8]; and d) is a major threat to the sustained increase of life expectancy [9].

The psychosocial consequences represent the most prevalent co-morbidities associated with obesity [10]. Obese young people have low self-esteem (SE) and a reduced quality of life (QoL), mainly associated with their perceptions of physical appearance, athletic skills and social functioning [6]. There seems to be a dose–response association between the Body Mass Index (BMI) and a worse health related QoL in such a way that severely obese people have significantly higher values than those seen in people with a less severe degree of obesity [11]. Moreover, obese individuals show higher levels of body dissatisfaction [12] and higher levels of depression [13]. Body Image (BI) has been consistently identified as a protection factor against the development of risky behaviour associated with obesity [14].

Obese adolescents with low levels of self-esteem have shown significantly higher levels of sadness, loneliness and anxiety and are more susceptible to engage in experimental/risky behaviours [9], such as smoking or drinking [15]. Fonseca and Matos [16] showed that overweight adolescents find it harder to make new friends when compared with their peers who are not overweight.

The questions of physical fitness associated with health are increasingly determinant in the way young people negotiate their daily lives and prepare for the future. In adolescents, a low cardiorespiratory fitness value is closely associated with an increased risk for developing cardiovascular diseases, regardless of country, age or gender [17]. To avoid the accumulation of risk factors for the development of cardiovascular diseases, the *European Youth Heart Study*[18] recommends that young people should perform more than one hour of moderate to vigorous physical activity (MVPA) per day. It has further been identified that higher amounts of MVPA hours are associated with a decrease in cardiometabolic risk factors [19]. However, an increase in physical activity (PA) alone does not solve the problem, as a decrease in sedentary behaviours (e.g. screen time) is crucial metabolic risk and adiposity control [20].

The health of individuals does not come only from their biology and/or individual actions but also from the biology and actions of those around them [21]. This issue is particularly relevant in adolescence as friends play an essential role in the developmental process, through the emotional support that is associated with social affection. Very often, peers are referred to as a barrier to regular PA and healthy diet [22]. More studies are needed to better understand these reciprocal influences, in order to be able to deal in a more effective way with the psychosocial factors negatively impacting the life of the obese adolescent [23]. Interventions that are intended to improve self-efficacy and social functioning in young people can bring specific benefits to obese adolescents [24]. However, and despite different authors e.g. [25], having suggested that an increase support from peers in contexts of PA implementation may constitute a viable way of promoting active lifestyles among young people, this area has still not been much studied.

Although the literature consistently points out the direction for solving the problem, it has generally not been possible to directly and effectively influence PA [26]. Interventions using mediators have shown effective results in changing behaviour in the short term and there is increasing evidence that this approach may have long-term effects [27]. The role of self-efficacy has been clearly pointed to as a mediator in the relationship between the intervention and PA [28]. Recently, Rhodes and Pfaeffli [29], in a wide-reaching review, suggested that changes in the structure of self-regulation might have a greater mediator effect on changing behaviour relative to PA.

Our program, named **T**ratamento da **O**besidade **P**ediátrica (TOP), standing for Paediatric Obesity Treatment, has been implemented in 2004, and is the result of the cooperation between the Paediatric Obesity Clinic, Department of Paediatrics, at Hospital de Santa Maria (HSM) and the Faculty of Physical Education and Sport at Universidade Lusófona (UL) in Lisbon. This initiative gave rise to an innovative, yet sustained programme where several disciplines including PA specialists are simultaneously present in the Clinic, making it possible that adolescents receive integrated medical, nutritional and PA care on the same day. Respecting the most recent recommendations [30], we use behaviour modifying techniques aimed not only at the adolescents but also at their families and peers, using techniques based on the Self Determination Theory (SDT) and Motivational Interviewing (MI) [31] within a treatment protocol that has been presented at the Society for Adolescent Health and Medicine [32]. Based on our previous experience and on recent literature, it was theorised that to provide better care it would be necessary: a) more extensive and frequent contact with the treatment team and b) the inclusion of peers to assist in the weight control tasks. The literature shows that an adolescent’s health behaviour is associated with that of their peers [23], through social contagion [33,34] mechanisms. The reasoning behind the design of this project is the belief that providing regular PA, interactive sessions (IS) [35] and specific moments of concentrated intervention – holiday camps [36], would offer the right context for both reaching a more extensive contact with the treatment team and allow for the inclusion of peers in the treatment. This intervention is grounded on the rationale presented and the health behaviour modification model, based on the Self-Determination Theory - SDT [37]. The team believes that this interaction between PA, positive psychological effects and reduction in BMI will lead to an increase in the perceived well-being of adolescents, which in turn will reinforce health behaviour, as has been seen with adults [38].

### Objective

The main objective of this project is the development, implementation and assessment of an adolescent obesity treatment programme, which will use PA and interactive sessions to promote skills for achieving weight control in a context of increased contact between the adolescents, peers and the team.

As such, the following objectives were defined:

1. To contribute to an effective weight management of adolescents, through reducing the BMI z-score and% Fat Mass (FM), increasing PA and reducing sedentary behaviour;

2. To analyse the effects of including peers and an increased exposure time of adolescents to PA and IS, on psychosocial health, eating habits, physical fitness and metabolic and inflammatory markers.

## Methods/design

### Study design and setting

The study is experimental and will be based on a Non-Randomised, non-blinded Controlled Trial including two groups: a) A Comparison group, which will receive the standard treatment at the clinical setting, including medical assessment, dietary and PA counselling every 3 months, with access to a 12-month programme including weekly IS and PA sessions and holiday camps; b) An experimental group which, in addition to the standard treatment, will have access, together with a peer of their choice, to a 12-month programme including weekly IS and PA sessions and holiday camps. The adolescents will be allocated to the groups according to the existence or not of peers of their choice to escort them in the intervention.

**Figure 1:**
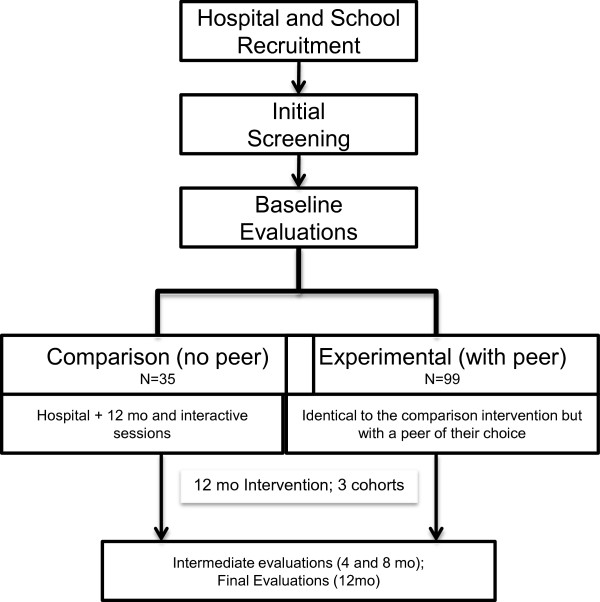
Study design.

The setting of the study will be an Exercise and Health University Study Centre and the Paediatric Obesity Outpatient Clinic at Hospital Santa Maria (HSM). All participants will be recruited in the Lisbon region, Portugal (Figure 1).

### Ethics approval and registration of the trial

The Ethics Committee of Faculdade de Medicina de Lisboa, University of Lisbon, approved this study, with the number 092/2013. Written consent will be obtained from both the adolescents and their parents or legal care providers. The study is registered in clinicaltrials.gov with the number NCT02024061.

### Participants

The participants will be randomly selected from two origins: the Paediatric Obesity Clinic at HSM and schools in the Lisbon region. Adolescents from the schools will be identified as obese by their Physical Education teacher based on their BMI percentile [39], and referred to the HSM Clinic for further assessment before being included in the study.

### Sample size calculations

To calculate the sample size we focused on the primary outcomes, using two-tailed tests (p < 0.05), for a power of 0.90. For the projected sample (134), we estimate that the analyses will have a power to detect effect sizes of 0.25. Specifically, we estimate that our groups should be made up of: 99 participants, divided into 3 cohorts of 33 participants – experimental group; and two cohorts of 12 participants and one of 13, in the comparison group. We are aware that a lengthy intervention may result in an attrition rate of about 25%, so we are expecting a drop to 0.82 in the study’ power, if this attrition is observed.

### Eligibility criteria

Inclusion Criteria: Obese adolescents with a BMI greater than or equal to the 95^th^ percentile [39], aged between 14 and 17, with Caucasian origin, agreeing with the commitment.

Exclusion criteria: a) cognitive impairment; b) pregnancy; c) serious illnesses; d) other factors with contraindication for regular PA.

### Subject withdrawal

The following will give rise to the withdrawal of participants:

•Attendance less than 75% of the IS or PA sessions;

•Acute illness implying non-compliance with the planned sessions;

To ensure that the participants comply with the assumptions, records will be kept of the attendance at the IS and PA sessions. Participants will not be substituted.

### Intervention

The study comprises an experimental group and a standard treatment group. The intervention will be similar in both groups with the exception that in the experimental group every adolescent will be escorted by a peer of their choice at every moment of the intervention (IS, PA sessions and holiday camps). The multidisciplinary team is made up of physical activity specialists, nutritionists/dieticians, psychologists and paediatricians, all of them with previous training in adolescent obesity, resulting from their involvement in the Paediatric Obesity Clinic at HSM.

### Interactive sessions

Both the comparison and experimental groups will meet the intervention team once a week. These meetings are conducted with both groups, with the intervention being identical in its contents, since the differential factor is the presence or absence of the peer of choice. The joined session will last for about 120 minutes, covering contents on PA/exercise, nutrition and behavioural change in a context of small activity groups (including peers), using an experiential learning methodology, which consists of increasing one’s skills through transforming the experience [35].

The main objective is to bring the participants closer to independent decision making on their wish to make changes about their lifestyle and weight status (and how to do so), and subsequently help them to face the consequences of their choices, regardless of the results.

The participants are encouraged to discover situations in their daily lives that can be changed in order to increase the number of calories burnt by more than 2000 Kcal per week [39]. This can be achieved more formally (physical exercise in a guided context) or informally (PA). The University offered an exercise room that is available for daily use by the participants. Each participant will be given a pedometer to help monitor the number of steps taken each day. The use of pedometers has been associated with significant increases in PA and significant reductions in BMI [40].

The intervention is based on a group functioning rationale which, in accordance with the social contagion theory [33], may contribute to positively influence the different members of the group. Peer group influence is major at this age and it is expected that young people will be more active when they are accompanied by their peers and their friends [41]. Benefits arising from group work, in the context of obesity management, were obtained through improving social functioning, which was positively associated in dimensions related with self-concept [34]. This effect was also seen in a recent study [42], which was intended to assess a PA intervention based on the use of pedometers and provision of support material to peers. It has been shown that the best results were obtained in the group that received only peer support material and objectives to be reached with the use of the pedometers. Salvy and colleagues [41], in a study with 88 adolescents, showed that the presence of a friend increased the motivation to be physically active. The use of peer and/or family support material has been suggested as an effective mean of promoting PA in young people at risk of sedentary behaviours [43], both at the beginning of the maintenance phase [44], and as an ongoing source of support for improving psychosocial functioning [45].

The IS included a variety of challenging physical activities, support provision for definition of a structured training plan, adjustment of daily tasks according to an active lifestyle, reduction/modification of sedentary behaviour strategies (e.g. reduction of screen time), help in discovering the preferred type of activity overcoming some myths and barriers around exercise/PA, and support in ambivalent behaviour. Because PA is the main modifiable component associated with energy expenditure in the energy balance equation [39], various international organisations have looked at PA promotion as a crucial modifiable health behaviour [46].

In addition to encouraging PA, it is equally important to reduce/modify sedentary behaviour [39,47]. Tremblay et al. [48], in a systematic literature review, showed that over 2 hours of television a day was associated with an unfavourable body composition, less physical aptitude, lower self-esteem values and more maladjusted social behaviour. According to the Canadian Sedentary Behaviour Guidelines for Children (5-11 y) and Youth (12-17 y) recently launched, it is recommended that for health reasons, children and adolescents should reduce daily sedentary time, by means of limiting screen time to a maximum of 2 hours a day, avoiding motorised transportation, along with the reduction of sitting time and time spent “inside the house” [49].

The IS also seek to provide self-regulation strategies for diet/nutrition behaviour improvement through providing information focused on increased awareness about nutritional facts; negotiating the modification of dietary patterns [50]; and using the food traffic light [51]. According to the recommendations of Spear et al. [39] and Davis et al. [52], there will be a focus on learning specific strategies/skills which include the importance of breakfast; increasing the number of meals during the day; avoiding hunger and unregulated consumption; reducing emotional (compulsive) and/or distracted eating; eating according to energy needs; consumption of food with a high satiety index; reduction in food consumption rich in animal fat; increase in the consumption of fruit, vegetables, cereals and other fibre rich foods; reduction in sugar consumption; limited consumption of drinks and fast food; lower portions size; and learning how to read and understand food labels.

With regard to cognitive-behavioural modification, the focus will be on the identification of problems and face the difficulties that arise during the programme. As there is nothing more central to behaviour than motivation, the whole interaction will be self-motivation and self-regulation centred, seeking to identify personal resistance and barriers, developing skills for prevention, identification and overcome [52]. Specific strategies [39] will include: the increase in self-efficacy through analysing and overcoming typical barriers such as lack of time, skill or accessibility; making planning more flexible; positively accepting failures, and/or that change is neither quick nor easy; building a system of incentives; and defining realistic, tangible objectives.

### Physical activity intervention

The physical activity intervention will comprise a weekly exercise session, taking place on the same day of the interactive session, and the use of pedometers to promote the increment of PA during the rest of the week. To progressively reach the 60 minutes of MVPA, we will aim that adolescents take 10000 to 11700 steps every day by the end of the intervention [53], specifying individual plans depending on their initial physical activity stage.

The exercise sessions will comprise several types of activities, from group classes focusing on aerobic exercises, sports, walking or running periods, dance, to strength sessions. We intend to show an extended array of activities that can be then adopted by the participant. Each session is planned for 60 minutes providing moderate to vigorous intensities.

More specifically, the strength sessions are planned according to the initial assessment carried out according to the 10-RM test. The training load is estimated for 50% of theoretical 1-RM [54]. The training plan consists of a circuit with 8 exercises for strength training, alternating between the torso and lower limbs. There will be 2/3 series of moderate intensity (30″ activity alternated with a 30″ break) or vigorous intensity (45″ of activity alternated with a 15″ break), thus respecting all of the recommendations [55]. Moderate to vigorous cardiovascular exercises, from 50 to 85% of the HR Reserve [54] will also be included, with a total volume of 10 to 30 minutes, according to the choice of the participants. The intensity of each exercise will be monitored both through the use of a heart rate monitor and the use of the perceived exertion scale for young people (OMNIA-RES) [56].

### Discontinuation

If participants miss more than 25% of the sessions, they will be removed from the analysis. The intervention will be discontinued if more than 50% of participants give up.

### Data collection

The variables will be assessed according to Table 1.

**Table 1:** Variables, measures and timing of the data collection

**Parameters**	**Assessed through…**	**When**
IS attendance	Attendance sheets	IS
Frequency of exercise	Training plans and attendance records	IS and Exercise sessions
PA	Pedometers (OMRON Walking Style II, HJ-113-E) PA records (7-Day Physical Activity Recall)	IS/Holiday Camps
PA and Sedentary behaviour	Accelerometers (Actigraph GT3X)	0, 4, 8, 12, 24, 36 months
BMI	Weight (OMRON BF-500) SECA height stadiometer	
% Fat mass	Bioimpedance (OMRON BF-500)	
Body composition	Dual-energy X-ray absorptiometry, DEXA (QDR-1500; Hologic, Waltham, MA)	
Physical fitness	FITNESSGRAM test battery	
Caloric intake	Food records (4-day recall)	
Quality of Life	Impact of Weight on Quality of Life – Kids (IWQOL-kids) Kidscreen-27	
Well-being	Rosenberg Self-Esteem Scale (RSES) Body Image Assessment Questionnaire (BIA) Body Shape Questionnaire (BSQ)	
Peers	Perceptions of Parents Scales (POPS) – adapted to peers Self-Perception Profile for Adolescents (SPPA) – Social Acceptance Scales and “Best Friends”	
Self-regulation variables	Treatment Self-Regulation Scale (TRS) Locus of Causality for Exercise (LCE) Weigh Management Self-efficacy (WMSE) Behavioural Regulation in Exercise Questionnaire (BREQ-2) The Psychological Need Satisfaction in Exercise Scale (PNSE)	

Notes: IS – Interactive sessions; PA – Physical Activity.

### Outcomes

Taking into account the recommendations of Barlow [57] and following analysis of the results of two recent meta-analyses [1,5], the following outcomes were defined (see Table 2).

**Table 2:** Study outcomes

**Variables**	**Goal**
BMI	5% z-score reduction
Body composition	5% reduction of percent fat mass
Psychosocial	Improvements of 0.5 standard deviation
PA time	Increase moderate/vigorous PA (≥60 min per day)
Sedentary behaviour	≤ 90 minutes per day
Caloric intake	1000-1500 Kcal daily intake

Note: PA – Physical Activity.

### Data analysis

We plan to use repeated measures analysis of variance to evaluate the impact of the intervention. Additionally, we plan to use structural equation models and/or multiple mediation analyses to assess the presence of predictors of the results of the intervention [58].

The intention-to-treat principle (last observation carried forward) will be followed for handling the missing data.

## Discussion

The aetiology of paediatric obesity is influenced by biological, physiological, environmental and contextual factors [59], with human behaviour being the aggregator factor not only in obesity but in health in general [60]. This intervention is mainly intended for a better understanding of how behaviour can be modified.

Adolescence is that time in life where the ability to learn increases and new habits are adopted [61]. This issue is one of the strongest aspects of the intervention, which intends to enhance the perspective of positive health both based on the increasing individual autonomy and on the peer group interaction as a key element for understanding and potentiating various positive kinds of behaviours [62].

It is well known that only a small part of the populations undergoing interventions to lose weight actually succeed (and maintain) in the long term. Usually, as soon as the intervention ends, PA tends to decrease. As such, and as it is not possible to intervene directly in PA, where should we intervene? Research has shown how motivational processes may play a crucial role as mediators of behavioural change and maintenance [63]. Thøgersen-Ntoumani and Ntoumanis [64] showed the importance of encouraging self-determined motivation in order to improve the quality of the experience and the perpetuation of the behavioural change through exercise.

The sensation of choice or desire when facing a specific behaviour, places the individual in a privileged position in the motivation continuum [63], and can be manipulated through self-regulation. We believe this is a central question, given that SDT proposes that PA can be intrinsically motivated through increasing autonomy and skills development [63].

It is hoped that the results of this trial will contribute to increasing the knowledge on the different processes involved in the management of paediatric obesity (how, when, in which ways and why) and will enable to provide some answers that will for sure merit to be further explored.

## Competing interests

The authors declare that they have no competing interests.

## Authors’ contributions

HF and ALP designed the study and wrote the first draft of the manuscript. ALP, SCM and LF designed the intervention and decided upon the data collection methods. ALP and AQ were responsible for the data analysis decisions and sample size calculations. HF is the principal investigator of the study, reviewed the draft and wrote the final version of the manuscript. All authors read and approved the final manuscript.

## Pre-publication history

The pre-publication history for this paper can be accessed here:

http://www.biomedcentral.com/1471-2431/14/89/prepub
